# Impact of a multi-level, multi-component intervention to improve elementary school physical education on student cardiorespiratory fitness: an application of the parametric g-formula

**DOI:** 10.21203/rs.3.rs-4331769/v1

**Published:** 2024-05-07

**Authors:** Hannah R. Thompson, Kristine A. Madsen, Caroline Nguyen, Thomas L. McKenzie, Sally Picciotto

**Affiliations:** University of California Berkeley; University of California Berkeley; University of California Berkeley; San Diego State University; University of California Berkeley

**Keywords:** cardiorespiratory fitness, adolescent health, physical fitness

## Abstract

**Background:**

School physical education is an important population-level health intervention for improving youth fitness. The purpose of this study is to determine the causal impact of New York City’s PE Works program on student cardiorespiratory fitness.

**Methods:**

This longitudinal study (2014–2019) includes 581 elementary schools (n=315,999 4^th^/5^th^-grade students; 84% non-white; 74% who qualify for free or reduced-price meals). We apply the parametric g-formula to address schools’ time-varying exposure to intervention components and time-varying confounding.

**Results:**

After four years of staggered PE Works implementation, 49.7% of students per school (95% CI: 42.6%, 54.2%) met age/sex-specific cardiorespiratory fitness standards. Had PE Works *not* been implemented, we estimate 45.7% (95% CI: 36.9%, 52.1%) would have met fitness standards. Had PE Works been fully implemented in *all* schools from the program’s inception, we estimate 57.4% (95% CI: 49.1%, 63.3%) would have met fitness standards. Adding a PE teacher, alone, had the largest impact (6.4% (95% CI: 1.0, 12.0) increase).

**Conclusion:**

PE Works, which included providing PE teachers, training for classroom teachers, and administrative/teacher support for PE, positively impacted student cardiorespiratory health. Mandating and funding multilevel, multicomponent PE programs is an important public health intervention to increase children’s cardiorespiratory fitness.

## BACKGROUND

The US Department of Health and Human Services recommends youth participate in at least 60 minutes of moderate-to-vigorous physical activity (MVPA) daily, specifically highlighting the importance of school-based physical activity opportunities to achieve this goal. ^1^ However, as of 2022, less than 25% of children aged 6–17 met the MVPA/day guideline. ^2^ Further, only 42% of US youths were considered physically fit, the lowest prevalence since estimates have been available, ^2,3^ which is expected to result in an estimated $1.7 trillion in lost productivity throughout their lifetimes. ^4–6^ Furthermore, racial/ethnic and income-related disparities in both physical activity and fitness levels will increase inequities in cardiovascular outcomes. ^5,7^

School physical education (PE) is an important population-level health intervention for increasing physical activity and improving youth fitness and has the potential to reduce health disparities.^8,9^ Almost all US states require elementary schools to provide PE; however, few states offer the resources and support to ensure the implementation of that legislation.^10^ Subsequently, compliance with state PE laws is extremely low.^11–13^Additionally, unequal provision of PE contributes to race/ethnic- and income-related health disparities.^14,15^

Elementary schools are less likely than middle and high schools to comply with PE state standards,^13^ making elementary schools a key target for interventions that increase compliance with existing PE laws. Qualitative evidence from educators suggests that interventions to increase PE-law adherence are more successful when they include an implementation process engaging multiple levels of influence (district, principals, and teachers) and provide tangible supports for PE, such as administrative guidance, credentialed PE teachers, and PE-related trainings.^16–18^ However, evidence about multilevel approaches for improving PE is lacking.

In 2015, less than 5% of New York City elementary schools were compliant with state law mandating at least 120 minutes of PE per week taught by a certified PE teacher.^19^ To address this, the Department of Education (NYCDOE), the nation’s largest school district and among the most racially/ethnically diverse,^20^ implemented PE Works, a multilevel intervention to improve PE.^19^ PE Works sought to remove historical systems-level barriers to PE implementation (e.g. limited funding, priority, and expectations for PE) by employing several evidence-based interventions^21^ simultaneously at both district and school levels. PE Works included: 1) a PE audit and feedback system^12^ combined with coaching to help schools with PE implementation and ensure PE teachers had appropriate training/support; 2) the provision of state-certified PE teachers in elementary schools; and 3) increased classroom teacher training to supplement PE.^22^ PE Works was implemented from school years 2015/16 through 2018/19, with intervention components rolled out in a time-varying fashion across the city’s elementary schools. While PE Works has the potential to serve as a national model for elementary PE, the effect of this approach on objectively measured student physical fitness has yet to be evaluated.

The purpose of this study is to determine the causal impact of a multi-level, multi-component approach to improve elementary PE on student cardiorespiratory fitness at the school level. We use data from 2014/15 through 2018/19 from 581 highly diverse NYCDOE elementary schools that participated in PE Works. We apply the parametric g-formula to address both schools’ time-varying intervention component exposure and time-varying confounding.

## METHODS

### Data Sources and Population

This longitudinal study spans school years 2014/15 through 2018/19 and draws data from: 1) NYCDOE Office of School Wellness Programs (OSWP) PE Works implementation dataset; 2) the NYC FITNESSGRAM ^®^ dataset jointly managed by NYCDOE and NYC Department of Health and Mental Hygiene (NYCDOHMH);^23^ and 3) publicly available school-level demographic and staffing data managed by NYCDOE.^24^ School inclusion criteria included: 1) elementary school serving students in grades K-5 (n = 663); 2) in a traditional education district (excludes 3 schools that were not required to administer the FITNESSGRAM^®^); and 3) having at least 3 study years of student cardiorespiratory FITNESSGRAM^®^ data (excludes 79 more schools). A total of 581 schools were eligible for inclusion. Study procedures were approved by UC Berkeley’s Committee for the Protection of Human Subjects (#202009 − 13643) and NYCDOE’s Institutional Review Board (#3788).

### PE Works Intervention

PE Works was implemented from 2015/16–2018/19 by the Office of School Wellness Programs (OSWP), which used an internal system to track implementation. The first component was PE audit and feedback^12^ combined with coaching. The PE Works audit consisted of 9 yes/no PE indicators, including those related to 1) PE teachers and instruction; 2) family-community ties; and 3) supportive environments. OSWP employees completed an audit through visual assessment and discussion with school administrators and the PE teacher, if available. OSWP personnel then created a feedback report detailing indicators needing improvement, with suggestions on how to improve. Feedback was shared with the school principal via tracked email requiring the principal’s electronic signature for receipt. During follow-up meetings, emails, and/or phone calls, OWSP personnel provided direct PE-related coaching and tracked the number of such interactions they had with each school. If a school made improvements, the number of indicators changed was recorded.

The second primary component was the provision of a state-certified PE teacher in elementary schools. Before PE Works began, only 10% of elementary PE classes were taught by a full-time certified PE teacher.^25^ Publicly available data indicated when a new PE teacher was funded in each school (https://infohub.nyced.org).

The third primary component was classroom teacher training in Move-to-Improve,^22^ NYCDOE’s classroom-based physical activity program designed to supplement PE minutes, with activities supporting State PE Learning Standards and aligned with core curriculum content areas.^26^ Classroom teachers were trained annually in Move-to-Improve through PE teacher-led workshops. A school is considered a Move-to-Improve All-Star school each year if at least 85% of teachers in the school are trained in Move-to-Improve.

Due primarily to NYCDOE’s size, implementation of PE Works intervention components were staggered across elementary schools (Table 1). In Year 1 (2015/16), the program was piloted in a cohort of 50 schools, which were purposely selected based on prior low compliance with state PE law and school characteristics associated with lower-quality PE provision (high proportion of students of color and students who qualify for free or reduced-price meals). The program was then rolled out to the city’s remaining elementary schools across program Years 2 through 4, based on OSWP capacity, to cohorts 2 and 3. By Year 4 (2018/19), 99% of sample schools had received their Audit, 77% their Feedback and coaching, 82% their PE Works-provided PE teacher, and 77% had Move-to-Improve All-Star status.

### Data

#### PE Works Data

OSWP provided data on the timing of the implementation of all PE Works components. Due to different implementation dates, the audit, feedback, and coaching component was split into two analytic variables. The school year a school received its PE audit, the audit variable changed from 0 (no) to 1 (yes) and remained 1 until the end of the study. Similarly, the school year a school received its PE feedback report, the feedback and coaching variable changed from 0 to 1, and once a school received a PE teacher, the PE teacher variable changed from 0 to 1. Move-to-Improve All-Star status could change each study year.

OSWP-provided data also included: PE Works cohort (1, 2, or 3); the number of PE teachers at the school at baseline; the number of conditions met after the audit (0–9); the number of audit conditions changed after OSWP coaching (0–9); and the number of OSWP coaching interactions with each school.

### Student Cardiorespiratory Fitness

The NYC FITNESSGRAM^®^ is administered annually by formally trained physical education and classroom teachers^23^ using district-provided equipment. NYCDOE schools are required to have at least 85% of eligible students complete the FITNESSGRAM^®^ annually. Student aerobic capacity is assessed by the Progressive Aerobic Cardiovascular Endurance Run (PACER test). For each study year, test results and the total number of 4th /5th -grade students tested overall and by demographic subgroups of interest (by sex, race/ethnicity, and FRPM status), were obtained from the NYC FITNESSGRAM^®^ dataset. The primary outcome was the annual school-level proportion of 4th /5th -grade students who met performance criteria for aerobic capacity using the sex- and age-specific Healthy Fitness Zones (HFZ) for aerobic capacity set forth by the Cooper Institute (the developer of the FITNESSGRAM^®^),^27^ which provides an indication of present and future cardiorespiratory health (hereafter called meeting fitness standards for aerobic capacity).

### School and Student demographics

Publicly available school-level data were downloaded from NYCDOE’s data website (https://infohub.nyced.org) for each study year, including total school enrollment, student enrollment by race/ethnicity (Asian, African-American, Latino/a, and White), and proportion of students eligible for free or reduced-price meals (FRPM: a proxy for socioeconomic status).

### Statistical Analysis

The parametric g-formula is a generalization of epidemiologic standardization to longitudinal data.^28,29^ It estimates the effects of hypothetical interventions, and unlike traditional regression, it adjusts correctly for time-varying confounding, even when covariates are affected by prior “treatment.” In this case, the interventions considered were: (a) no PE Works (i.e., all components set to 0 in all 4 years for all schools), (b) immediate implementation of all PE Works components (i.e., all components set to 1 in all years for all schools), immediate implementation of hiring a dedicated PE teacher but no other PE Works components, and immediate implementation of Move-to-Improve training but no other PE Works components.

Applying the parametric g-formula occurs in several steps. In Step 1, the observed outcome, “treatments”, and time-varying covariates are modeled, saving the estimated coefficients. Next, a Monte Carlo sample is drawn from the baseline data (school year 2014/15). A covariate history is generated for each school in the sample by predicting, for each subsequent school year, values for all time-varying covariates (including the sociodemographic composition of the school, implementation of PE Works components, and proportion of students meeting aerobic capacity fitness standards) on the basis of prior values of these covariates and the coefficients estimated in step 1. This procedure is repeated under interventions on the PE Works components. For example, a counterfactual covariate history corresponding to “no PE Works components implemented” is generated by setting all PE Works components to zero at every year and using those values, the estimated coefficients from Step 1, and the other predicted covariates to predict each covariate under that scenario. We can then compare the expected proportion of students meeting fitness standards if all schools had received implementation of all PE Works components starting immediately in 2015/16 to what would have happened if PE Works had not been delivered at all.

In this application, the following variables were modeled for each school in each year: proportion of students meeting aerobic capacity fitness standards (outcome); all four of the PE Works components (primary predictors); total school enrollment; proportion of students who qualified for FRPM; the proportion of White students; the total number of students tested; the number of PE teachers at the school; and the number of days of PE per week. Each of these depended on a relevant subset of baseline and time-varying covariates (Supplement). The school-level proportion of students meeting fitness standards each year was modeled as a function of its value in the previous years; PE Works cohort; total number of PE teachers; number of audit conditions met; number of audit conditions changed after OSWP coaching; the number of total OSWP coaching interactions; Move-to-Improve All-Star status (both current and prior year); total school enrollment; proportion of students qualified for FRPM; proportion of White students; number of students tested; and the number of days of PE per week.

Separate analyses were run to test for effect modification by student sex, race/ethnicity, and FRPM status and to estimate the impact of PE Works in these subpopulations of students. Confidence intervals were generated nonparametrically using 500 bootstrap samples. Descriptive statistics were calculated in Stata (MP/16.1); the g-formula analyses were carried out in RStudio (2023.12.1) using the gfoRmula package (https://github.com/CausalInference/gfoRmula).

## RESULTS

The final analytic sample included 581 schools, with a total of 315,999 students contributing 558,598 student-year observations over the 5-year study period. At baseline (2014/15), the average enrollment was 647 students/school, with an average 48.8% female, 14.4% Asian, 26.7% African American, 40.9% Hispanic/Latinx, and 16.0% White students per school (Table 2). On average, 73.7% of students qualified for FRPM.

At baseline, on average, 196 students (95.2% of those eligible) underwent fitness testing per school. At the school level, an average of 40.1% of students were meeting aerobic capacity fitness standards. The school-level average for meeting aerobic capacity fitness standards was higher for males (44.9%) than females (35.4%); for Asian (43.1%) and White (40.7%) than for African American (38.4%) and Hispanic/Latino (38.4%) students; and for students who did not qualify for FRPM (42.0%) compared with those who did (38.4%).

Table 3 presents the predicted school-level proportion of students meeting aerobic capacity fitness standards under the observed PE Works components implementation across *all* 4 years (2015/16–2018/19) compared to: 1) what would have happened had PE Works not been implemented, and 2) under a hypothetical PE Works intervention in which *all* schools received *all* 4 primary PE Works components (PE needs assessment/audit, feedback, PE teacher provision, and increased classroom teacher training in Move-to-Improve) for *all* 4 years. Under observed PE Works conditions, by the final year (2018/19), 49.7% of students (95% CI: 42.6%, 54.2%) met aerobic capacity fitness standards. Had PE Works not been implemented, we estimate 45.7% (95% CI: 36.9%, 52.1%) would have met aerobic capacity fitness standards (difference of −4.0 percentage-points (95% CI: 0.3%, 8.1%) from observed PE Works implementation). Had PE Works been fully implemented in *all* schools from the beginning of Year 1, we estimate 57.4% (95% CI: 49.1%, 63.3%) of students would have met aerobic capacity fitness standards (difference of 11.7% percentage-points (95% CI: 3.8, 19.2) from *no* PE Works implementation).

There was no formal evidence of effect modification (male vs. female, p = 0.917); race/ethnicity (Asian vs. African American, p = 0.932; Asian vs. Hispanic/Latino, p = 0.595; Asian vs. White, p = 0.994; African American vs. Hispanic/Latino, p = 0.656; African American vs. White, p = 0.926; Hispanic/Latino vs. White, p = 0.590); and FRPM status (qualifies vs. does not qualify, p = 0.682; Supplement). However, we present stratified models (Table 3), as differences in outcomes by student demographic characteristics are of consistent interest to NYCDOE and researchers.

Figure 1 shows the observed and predicted proportions of all students meeting aerobic capacity fitness standards for each school year. Figures stratified by student sex, race/ethnicity, and FRPM status can be found in the Supplementary Materials.

We additionally examined the estimated effect of each PE Works component individually. Compared to PE Works not being implemented, adding a PE teacher, alone, resulted in an estimated 6.4 percentage-point (95% CI: 1.0, 12.0) increase in the school-level proportion of students meeting aerobic capacity fitness standards after 4 years. Move-to-Improve All-Star status, alone, resulted in an estimated 5.3 percentage-point (95% CI: 0.3%, 10.6%) increase. Neither the PE audit, alone, or the Feedback/Action Plan, alone, nor the Audit and Feedback combined, resulted in a statistically significant estimated increase in the proportion of students meeting aerobic capacity fitness standards.

## DISCUSSION

This is the first known study to capitalize on a large, natural experiment to examine the causal impact of a multi-level, multi-component PE intervention on elementary students’ cardiorespiratory fitness. NYCDOE’s PE Works program, intentionally designed to address low PE provision and improve student health, had a positive impact on cardiorespiratory fitness across student groups after four years of varying implementation across 581 schools. This study contributes to the evidence base demonstrating PE’s contributions to improvements in students’ aerobic fitness.^30^

A major strength of this study is the application of the parametric g-formula, which allowed for a comparison of what would have occurred *without* PE Works to what would have occurred had PE Works been implemented in *all* schools from the *beginning* of the program, and determined the causal impact of a non-randomized natural experiment. Had all schools received PE Works from Day 1, we estimate a 12% increase in the proportion of students meeting aerobic capacity fitness standards after four years, compared to had PE Works not been implemented. This translates to improved cardiorespiratory fitness for nearly 40,000 students, representing large public health impact. While implementation in all schools from day 1 was not realistic in NYCDOE, smaller districts could more feasibly provide PE teachers and an evidence-based curriculum (like Move-to-Improve) across all schools, with potentially comparable impact, using lessons learned from NYCDOE.^31^ Testing this method in financially-able school districts would help further inform scalability and generalizability.

While PE Works did not reduce cardiovascular disparities, it did not increase them (as other well-intentioned health policies have);^32^ the program resulted in improved aerobic capacity for students *across* groups. While formal tests for effect modification did not yield statistically significant results, stratified models signified a particularly strong impact for both sexes, Latino students, and students qualifying for FRPM. While formal evidence of a reduction in fitness disparities was hoped for, improving fitness for all students is still far preferable to leaving certain students behind.

This work represents a real-world program, highlighting efforts driven and executed by the largest public school district in the US, rather than by researchers. The program’s first year was funded by an unprecedented Mayoral initiative, which provided $6M to pilot PE Works in 185 elementary schools, with 50 schools adding a credentialed PE teacher.^43^ Data from the pilot year offered valuable insight into the specific challenges schools faced in providing PE and informed Year 2–4 implementation, which occurred after the city invested significant additional funding ($100M) for citywide expansion.

This massive cash infusion is not easily replicated in other school districts; thus understanding the singular impact of each primary PE Works intervention component is important. Adding a PE teacher, alone, resulted in an estimated 6% increase in the proportion of students meeting aerobic capacity fitness standards. Before PE Works began, only 10% of elementary PE classes were taught by a full-time credentialed PE teacher.^70^ Classroom teachers, whose multi-subject credential usually involves only a few hours of PE-specific education, are less well-equipped to deliver PE than credentialed PE teachers, who have at least a year of PE-specific training.^61,71^ Other studies support this finding, demonstrating that credentialed PE teachers are associated with greater amounts of PE,^72^ more daily MVPA,^73^ and better student cardiorespiratory fitness.^12^

Increased classroom teacher training in an evidence-based program also led to improved student fitness. Had all schools had Move-to-Improve All Star Status from Day 1, we estimate a resulting 5% increase in the school-level proportion of students meeting fitness standards. This finding is substantiated by prior evidence, with widely disseminated evidence-based PE programs (e.g. CATCH^33^ and SPARK^34^) demonstrating increases in student physical activity and fitness. Given the typically lower cost of programs that support elementary classroom teachers to lead PE, investing in programs like Move-to-Improve could be a sound alternative in the absence PE teacher funding.

While audit, feedback, and coaching alone did not impact student cardiorespiratory fitness, qualitative evidence from NYCDOE demonstrated the critical import of the support (including resources tailored to a school’s individual needs based on trusting district-school relationships) that audit, feedback, and coaching provided as part of this program.^31^ As this is a less expensive intervention than adding PE teachers or Move-to-Improve, it is important to test this approach. Work is currently underway to examine the impact of audit, feedback, and coaching alone on student health in Oakland, California elementary, with forthcoming results expected to contribute additional evidence.

The size and scope of PE Works makes it challenging to compare it to other interventions. Texas’s $37 million, 5-year, Texas Fitness Now program, a large (though less structured) investment, demonstrated no impact on student cardiorespiratory fitness. ^35^ However, it focused on middle schools, where PE is typically already block-scheduled and taught by credentialed PE teachers. In addition, middle schoolers may have already formed physical activity habits that are harder to change. Confirming the impact of a similar program on elementary students in other locations/states is important.

Several limitations deserve mention. First, this research relied upon secondary, NYCDOE-collected data; we lacked detailed quantitative data on coaching exposure/dose and direct systematic observations of PE classes (to validate how often PE occurred). In addition, school-reported data on PE minutes lacked variability (over 75% of schools self-reported meeting the state PE minute law at baseline), precluding our ability to use law compliance as an outcome. Other research has demonstrated that schools overreport PE time when self-reporting compliance.^12,31^ Finally, NYCDOE is a large and highly diverse urban school district; findings from this study may not generalize to other school districts with different school, student, and staff characteristics.

## CONCLUSIONS

PE Works had a robust, positive causal impact on student cardiorespiratory fitness, with adding PE teachers into schools having the greatest singular impact on improved student aerobic capacity. Synergistic and comprehensive PE programming that includes the provision of PE teachers, PE training for classroom teachers, and administrative/teacher support for leading PE, can positively impact student cardiovascular health. Further, it is feasible to implement in a large, highly diverse, and heterogeneous school district. When multi-level approaches are not viable, adding and supporting PE teachers in elementary schools is a public health intervention worth investing in. Future evidence from other school districts will better illuminate the potential for less costly interventions, such as PE audits, feedback, and coaching to impact student health.

## Figures and Tables

**Table 1:** Number of elementary schools implementing PE Works’ primary intervention components, ^A^ 2014–15 through 2018–19 (total n = 581 schools)

PE Works					
	2014–15	2015–16	2016–17	2017–18	2018–19
Physical Education (PE) audit	N/A	163 (28%)	576 (99%)	577 (99%)	577 (99%)
PE feedback and coaching	N/A	0 (0%	204 (35%)	411 (71%)	411 (71%)
PE Works credentialed PE teacher	N/A	40 (7%)	78 (13%)	170 (60%)	477 (82%)
Move-to-Improve All-Star status	71 (12%)	105 (18%)	103 (18%)	211 (36%)	445 (77%)

A PE Works intervention components included: 1) a physical education (PE) needs assessment/audit; 2) feedback from the needs assessment in the form of an action plan, combined with coaching to help schools with PE implementation teacher training; 3) the provision of state-certified PE teachers in elementary schools; 3) 85% of classroom teachers trained in leading PE through the Move-to-Improve program. Note, unlike the other components, some schools received Move-to-Improve training before PE Works.

**Table 2:** New York City Department of Education elementary school sample baseline demographic characteristics, 2014–15 school year (n = 581 schools; n = 108,898 4th /5th -grade students tested via FITNESSGRAM^®^)

	Mean ± SD
School-level characteristics	
Enrollment, All Students	647 ± 318
% female students	48.8 ± 2.4
% Asian students	14.4 ± 20.1
% African American students	26.7 ± 28.1
% Hispanic/Latino students	40.9 ± 26.7
% White students	16.0 ± 22.6
% Other/multiple race/ethnicity students	2.0 ± 2.6
% of students who qualify for free or reduced-price meals	73.7 ± 23.3
Student-level characteristics	
Number of 4th /5th grade students tested via FITNESSGRAM^®^, per school	196 ± 109
Proportion of 4th /5th grade students tested for FITNESSGRAM^®^, per school	95.2 ± 12.4
School-level proportion of 4th /5th grade students who tested in the Healthy Fitness Zone ^A^ for aerobic capacity, All students	40.1 ± 24.8
Female	35.4 ± 25.1
Male	44.9 ± 25.3
Asian	43.1 ± 33.3
African American	38.4 ± 27.3
Hispanic/Latino	38.4 ± 25.0
White	40.7 ± 31.9
Students who qualify for free or reduced-price meals	38.4 ± 24.4
Students who do not qualify for free or reduced-price meals	42.0 ± 28.0

A The FITNESSGRAM, uses Healthy Fitness Zones to evaluate students’ fitness performance. These zones are criterion-referenced standards and represent minimum levels of fitness for age and sex that offer protection against the diseases that result from sedentary living. Aerobic capacity reflects the maximum rate of oxygen uptake and use during exercise.

**Table 3:** Predicted school-level proportion of students meeting aerobic capacity fitness standards in 2018/19 (final year of PE Works) under: (A) the observed PE Works implementation across all 4 intervention years (2015/16–2018/19), compared to what would have happened had (B) PE Works not been implemented and (C) all schools received all PE Works components ^A^ for all 4 intervention years (n = 581 elementary schools)

	A. Mean under observed PE Works components (95% CI)	B. Estimated mean under no PE Works components (95% CI)	C. Estimated mean under all/complete PE Works components (95% CI)	Mean difference observed vs. no PE Works components (A vs. B) (95% CI)	Mean difference no vs. all/complete PE Works components (B vs. C) (95% CI)
All students	49.7 (42.617, 54.176)	45.7 (36.923, 52.056)	57.4 (49.143, 63.336)	4.0 (0.307, 8.081)	11.7 (3.812, 19.179)
Female	50.0 (43.236, 54.640)	46.7 (37.888, 52.883)	57.6 (50.094, 62.859)	3.4 (−0.468, 7.742)	10.9 (3.646, 19.199)
Male	52.2 (45.223, 56.934)	47.8 (38.731, 54.800)	59.7 (52.063, 65.445)	4.4 (0.508, 8.584)	11.9 (4.019, 19.623)
African American	49.6 (43.031, 55.745)	48.7 (39.158, 57.158)	54.2 (46.709, 61.725)	0.9 (−3.710, 6.177)	5.5 (−2.633, 14.014)
Asian	40.3 (30.006, 51.407)	37.1 (23.937, 50.302)	44.2 (34.242, 56.122)	3.2 (−3.010, 10.224)	7.1 (−3.087, 18.833)
Hispanic/Latino	46.7 (40.496, 52.284)	42.8 (34.296, 50.014)	54.1 (46.966, 61.011)	3.9 (0.063, 8.227)	11.3 (3.904, 18.999)
White	50.9 (43.931, 56.998)	49.4 (39.524, 57.606)	56.0 (48.381, 63.336)	1.5 (−3.311, 6.848)	6.7 (−1.387, 15.401)
Eligible for free or reduced-price meals	47.5 (41.563, 53.375)	43.6 (35.935, 51.251)	54.9 (47.821, 62.344)	3.9 (0.030, 8.104)	11.3 (3.791, 19.806)
Not eligible for free or reduced-price meals	50.0 (41.825, 57.783)	51.6 (41.157, 61.478)	55.2 (45.776, 64.902)	−1.6 (−6.797, 4.422)	3.5 (−5.945, 14.474)

A PE Works intervention components included: 1) a physical education (PE) needs assessment/audit; 2) feedback from the needs assessment in the form of an action plan, combined with coaching to help schools with PE implementation teacher training; 3) the provision of state-certified PE teachers in elementary schools; 3) increased classroom teacher training in leading PE through the evidence-based Move-to-Improve program.

## Data Availability

The authors do not have permission to share data, but data could be made available through required IRB approval and data use agreement(s) with the New York City Department of Education.
